# Emerging Role of Aprepitant in Cannabis Hyperemesis Syndrome

**DOI:** 10.7759/cureus.4825

**Published:** 2019-06-04

**Authors:** Swetha Parvataneni, Lionel Varela, Sireesha M Vemuri-Reddy, Mandy L Maneval

**Affiliations:** 1 Internal Medicine, Geisinger Health System, Lewistown, USA; 2 Family Medicine, Geisinger Health System, Lewistown, USA

**Keywords:** cannabis hyperemesis syndrome, aprepitant

## Abstract

Cannabis hyperemesis syndrome (CHS) is a clinical syndrome associated with prolonged and regular cannabis use. CHS is characterized by recurrent episodes of intractable nausea and vomiting. Given the overlap with other medical conditions and the frequent delay in diagnosis, finding an effective anti-emetic regimen for symptomatic control of CHS can be challenging. We report a case study where aprepitant (Emend) was successfully used as an anti-emetic in the treatment of CHS when all other common anti-emetics failed.

## Introduction

Cannabis is the most commonly abused illicit drug [1]. Approximately 192 million people worldwide use cannabis at least once a year [2]. With the legalization of cannabis in certain states, the prevalence and dependence from cannabis use have increased exponentially [3].

CHS is a variant of cyclic vomiting syndrome associated with chronic cannabis use, which was first described by Allen et al. in 2004 [4]. Symptoms of CHS overlap significantly with conditions such as cyclic vomiting syndrome (CVS), psychogenic vomiting, viral gastroenteritis, functional dyspepsia, and bulimia nervosa [5]. Therefore, diagnosis of CHS can be challenging and is often delayed, resulting in more frequent emergency department (ED) visits for nausea and vomiting [6].

Multiple diagnostic criteria have been developed over the years to diagnose CHS, and in 2012, major and minor criteria for the diagnosis of CHS were first published [7-8]. In 2016, CHS was further categorized as a functional gastrointestinal (GI) disorder [9]. Even after adequate diagnosis, there is still limited evidence for symptomatic treatment of CHS. In this case report, we describe the use of aprepitant, an NK1 receptor antagonist, in the treatment of cannabis hyperemesis syndrome (CHS).

## Case presentation

A 30-year-old woman with a past medical history of asthma and chronic intermittent nausea and vomiting for four years presented to the ED with the same complaint. Her symptoms were accompanied by non-focal, intermittent, moderately severe abdominal pain that had progressed in severity, prompting her ED visit. She was previously started on omeprazole and promethazine with only minimal alleviation of her symptoms. She had been smoking marijuana a few times per week for four years. Physical examination was unremarkable except for epigastric tenderness to palpation. An extensive laboratory and imaging workup including liver function tests, pancreatic enzymes, heavy metal levels, esophagogastroduodenoscopy, hepatobiliary scan, and computed tomography (CT) scan of the abdomen and pelvis were unremarkable, except for her toxicology screen. This was positive for cannabis with a confirmatory test revealing high levels of 11-Nor-9-carboxy-Δ9-tetrahydrocannabinol (THC-COOH) (94.5 ng/mL).

Throughout hospitalization, she was kept nil per os (NPO) and treated with ondansetron, metoclopramide, prochlorperazine, omeprazole, ranitidine, promethazine, and haloperidol, but remained symptomatic. However, when aprepitant was started, she responded well, was able to tolerate food in gradual increments, and was discharged symptom-free. Recommendations were made to abstain from cannabinoid products and follow up with her primary care doctor.

## Discussion

Understanding the physiology and mechanism of action of cannabinoid receptors is necessary to better understand the effects of aprepitant. There are two types of cannabinoid receptors. CB1 receptors are located in the central nervous and gastrointestinal system, while CB2 receptors are found within the lymphoid tissue of ileum [10-11]. The anti-emetic properties of cannabinoids are due to interaction with CB1 receptors in the hypothalamus, while the pro-emetic properties are related to CB1 receptors in the gastrointestinal system.

Tetrahydrocannabinol (THC) is the active compound in cannabis responsible for the anti-emetic properties of cannabinoids. Studies suggest a good anti-emetic response in humans and THC has been utilized in oncology to relieve nausea in chemotherapy patients [12-13]. While THC is well known for its anti-emetic properties at low doses, it can cause CHS at higher doses with chronic use [14]. This may be secondary to accumulation of THC in adipose tissue with chronic use [15]. Chronically elevated levels of THC can lead to desensitization of CB1 receptors resulting in delayed gastric emptying and hyperemesis [16].

The treatment of CHS is classified into two phases: hyper-emetic phase and prevention of relapse [15]. In the hyper-emetic phase, patients present with dehydration requiring fluids and antiemetics for symptomatic relief. Currently utilized antiemetic therapies include dopamine antagonists, 5HT3 antagonists, antihistamines, anticholinergic, benzodiazepines, corticosteroids, capsaicin, and opiates. The mechanism of action and efficacy of these medications in CHS are similar and have been described in previous studies [14,17]. Modalities such as drug rehabilitation and cognitive behavioral therapy are also used in the prevention of relapse.

Our case report discusses the use of aprepitant in CHS for symptomatic relief. The role of NK1 receptor antagonist aprepitant has been studied in children with cyclic vomiting syndrome and chemotherapy-induced nausea and vomiting but has not been studied in CHS [18]. NK1 receptors and their endogenous ligand, substance P, are present in the brain stem nuclei of the dorsal vagal complex which regulates the vomiting reflex and neurochemical responses to stress. By blocking NK1 receptors and thereby blocking binding of substance P to NK1 receptors, aprepitant controls nausea and vomiting [19]. A similar mechanism of action has been described for topical capsaicin in CHS. By binding to TRPV1 receptors, topical capsaicin decreases the release of substance P from nerve endings, thereby decreasing nausea and vomiting in CHS. These studies suggest a role for substance P in the anti-emetic pathway [20].

## Conclusions

Aprepitant is an NK1 receptor antagonist that plays a significant role in the control of nausea and vomiting in CHS. Given the success of this medication in our patient, further studies are needed to evaluate the efficacy of aprepitant in CHS.
